# Exploring ceRNA mechanisms in COVID-19 mRNA vaccine-induced myocarditis: implications for future vaccine design

**DOI:** 10.3389/fimmu.2025.1674049

**Published:** 2025-10-22

**Authors:** Jing Wang, Xin-Yi Sun, Qian Gao, Min Fu, Mian Xiao, Ning Du, Xi-Yuan Ge

**Affiliations:** ^1^ Central Laboratory, Peking University School and Hospital of Stomatology, Beijing, China; ^2^ National Center for Stomatology & National Clinical Research Center for Oral Diseases & National Engineering Research Center of Oral Biomaterials and Digital Medical Devices, Beijing, China; ^3^ Beijing Key Laboratory of Digital Stomatology, Beijing, China; ^4^ Department of Oral and Maxillofacial Surgery, Peking University School and Hospital of Stomatology, Beijing, China

**Keywords:** COVID-19 mRNA vaccines, *in vitro* transcription mRNA, IL-6, ceRNA, myocarditis

## Abstract

**Introduction:**

The emergence of Coronavirus Disease 2019 (COVID-19) caused by severe acute respiratory syndrome coronavirus 2 (SARS-CoV-2) necessitated the rapid development of effective vaccines, with mRNA vaccines demonstrating high efficacy and accelerated production timelines. However, reports of myocarditis following mRNA vaccination have raised safety concerns, and the underlying molecular mechanisms remain poorly understood.

**Methods:**

Human AC16 cardiomyocytes were transfected with *in vitro* transcribed (IVT) COVID-19 mRNA modified with N1-methylpseudouridine (m1Ψ). A panel of inflammatory cytokines, including IL-6, was quantified using a Luminex liquid suspension chip. IL-6 expression was further validated at the mRNA level by RT-qPCR and at the protein level by ELISA. Cardiomyocyte apoptosis was assessed by flow cytometry. Myocardial injury biomarkers, specifically Creatine Kinase-MB (CK-MB) and cardiac troponin I (c-TnI) were measured by ELISA. The ceRNA interaction was investigated through RNA immunoprecipitation (RIP), and dual-luciferase reporter assays.

**Results:**

IVT mRNA elicited a robust inflammatory response in cardiomyocytes, markedly upregulating the proinflammatory cytokine IL-6 (~2-fold). Under inflammatory conditions, IVT mRNA further exacerbated IL-6 secretion (~2-fold) and increased cardiomyocyte apoptosis (~1.3-fold). Additionally, IVT mRNA significantly elevated the levels of CK-MB (~1.5-fold) and c-TnI (~2-fold). Mechanistically, IVT mRNA functions as a competing endogenous RNA (ceRNA) for hsa-let-7f-5p, alleviating its suppression of IL-6 mRNA, and enhancing inflammatory responses in AC16 cardiomyocytes.

**Discussion:**

This study elucidated a molecular mechanism linking COVID-19 mRNA vaccines to myocarditis and highlighted the ceRNA-mediated crosstalk between IVT mRNA and IL-6. These findings underscore the importance of avoiding critical microRNA binding sites in the design of next-generation mRNA vaccine sequences to improve safety.

## 1 Introduction

The global outbreak of Coronavirus Disease 2019 (COVID-19), caused by the severe acute respiratory syndrome coronavirus 2 (SARS-CoV-2), has necessitated the rapid development and deployment of effective vaccines. Among the various vaccine platforms, mRNA vaccines have emerged as a groundbreaking approach due to their swift development timeline and high demonstrated efficacy (1, 2). Notably, the Pfizer-BioNTech BNT162b2 vaccine has been widely administered and has played a pivotal role in controlling the pandemic (3). However, emerging evidence has highlighted potential adverse effects associated with these vaccines, particularly myocarditis, which has been less frequently reported with inactivated COVID-19 vaccines (4–7). Myocarditis, characterized by inflammation of the heart muscle has raised significant concerns due to its potential to cause severe cardiac complications (8). Following the COVID-19 pandemic in 2020, the global incidence of myocarditis rose significantly (9). Although the risk of myocarditis has increased after receiving the COVID-19 vaccine, it remains lower than the risk associated with SARS-CoV-2 infection (10). Epidemiological studies have established a correlation between COVID-19 mRNA vaccination and an increased incidence of myocarditis, particularly among young males (11–14). Despite these findings, the precise molecular and cellular mechanisms underlying this vaccine-related adverse reaction largely unclear, highlighting the urgent need for further investigation to improve the safety of current and next-generation mRNA vaccines.

To gain mechanistic insights, several recent studies have employed animal models to mimic vaccine exposure and investigate potential immunopathological outcomes. For instance, Li et al. reported that high-dose intravenous administration of a COVID-19 mRNA vaccine in BALB/c mice induced multifocal myocarditis and pericarditis, accompanied by elevated cardiac interleukin-6 (IL-6) and additional proinflammatory markers, indicative of a robust systemic inflammatory response (15). Likewise, Lee et al. demonstrated that in a chronic inflammatory murine model, mRNA vaccination enhanced cardiac expression of IL-6 and other inflammatory mediators, suggesting that individuals with pre-existing inflammatory conditions may be more vulnerable to vaccine-induced myocardial effects (16). These findings support the notion that mRNA vaccines may indirectly contribute to myocardial inflammation through immunological pathways. Among these pathways, aberrant regulation of inflammatory cytokines such as IL-6—a central mediator in inflammatory diseases—has received increasing attention in the context of vaccine-induced immune responses (17, 18). Furthermore, the emerging role of competing endogenous RNA (ceRNA) networks in regulating immune and inflammatory responses has provided new perspectives on post-transcriptional regulation in disease pathogenesis.

ceRNAs, including mRNAs, long non-coding RNAs, and circular RNAs, regulate gene expression by competitively binding to shared microRNAs (miRNAs), thereby modulating a variety of cellular processes (19). miRNAs, a class of conserved, small non-coding RNAs, function as key regulators by inhibiting the expression of target genes (20). Disruption of ceRNA networks can lead to imbalances in biological processes such as differentiation, apoptosis, and proliferation, contributing to various inflammatory diseases and cancers (21, 22). Specifically, exogenous mRNAs introduced by COVID-19 vaccines may act as ceRNAs, sponging endogenous miRNAs and thereby perturbing the regulatory balance of immune mediators. However, the role of ceRNA-mediated mechanisms in the context of mRNA vaccine-induced myocarditis remains largely unexplored. To address this, we employed a human cardiomyocyte cell line (AC16) transfected with *in vitro* transcribed (IVT) mRNA to model vaccine exposure and investigate the downstream molecular changes associated with myocardial inflammation. This *in vitro* approach allows a focused examination of potential ceRNA interactions and inflammatory signaling pathways triggered by mRNA exposure in cardiomyocytes.

## 2 Materials and methods

### 2.1 Cell culture

AC16 human cardiomyocytes were kindly provided by Wuhan Pricella Biotechnology Co., Ltd (Wuhan, China). Cells were cultured in DMEM/F12 medium (Procell, Wuhan, China) supplemented with 10% (*v*/*v*) fetal bovine serum (FBS; Procell, Wuhan, China), and maintained at 37 °C in a 5% CO_2_-enriched humidified environment.

### 2.2 Preparation of IVT mRNA (m1Ψ modified)

The pmRVac-Spike UTR plasmid was constructed by VectorBuilder Biotechnology (Guangzhou, China) using Gibson assembly technology. The mRNA sequence of the BNT162b2 COVID-19 mRNA vaccine produced by Pfizer-Biotech has been disclosed (23).

The SARS-CoV-2 spike glycoprotein coding sequence was inserted into the pmRVac vector using Gibson assembly to generate a DNA template for *in vitro* transcription (IVT). The pmRVac vector contains a T7 promoter sequence, a 5’ UTR, a 3’ UTR, a 110 nt segmented poly(A) tail and a SapI restriction enzyme site used for run-off transcription. The insertion site of SARS-CoV-2 spike glycoprotein coding sequence was between the UTRs. The Gibson Assembly reaction product was transformed into E. coli and plated on LB containing 50 μg/mL kanamycin. The resulting colonies were screened by PCR to identify positive insertion events. The PCR positive clones were evaluated further by restriction digestion and Sanger sequencing. The correct plasmid was cultured in LB media and purified using an Endotoxin-Free Plasmid Midiprep Kit (Sigma-Aldrich, Burlington, MA, USA). Plasmid DNA concentration and purity were measured on a UV-Vis spectrophotometer, and then linearized using SapI restriction enzyme.

To produce uncapped *in vitro* RNA transcripts, linear DNA template was mixed with T7 RNA polymerase, nucleotide triphosphates (NTPs) and magnesium-containing buffer to set up the *in vitro* transcription reaction. The reaction mixture was incubated at 37 °C for 2 hours. In the process of *in vitro* transcription, the natural uridine was replaced with N1-methylpseuduridine (m1Ψ). Cap1 mRNA was produced using vaccinia capping system. After denaturation of uncapped transcripts by heating, vaccinia capping enzyme, 2’-O methyltransferase, GTP, S-adenosyl methionine (SAM) and capping buffer were added to set up the capping reaction. The reaction mixture was incubated at 37 °C for 1 hour. Subsequently, template DNA was removed by DNase I treatment. Capped transcripts were further purified using magnetic beads. The isolated mRNA was eluted with an acidic buffer and stored at −80 °C. The mRNA concentration and purity were measured on a UV-Vis spectrophotometer. The mRNA integrity was confirmed by denaturing agarose gel electrophoresis, and the sequence identity was verified via mRNA sequencing.

### 2.3 *In vitro* mRNA transfection

AC16 cardiomyocytes were seeded in 12-well plates 18–24 h prior to transfection. At 60–80% confluence, cells were transfected with IVT mRNA (0.1 μg/mL) using Lipo8000™ transfection reagent (Beyotime, Shanghai, China) as per the manufacturer’s instructions. Cells were collected 24–48 h post-transfection for subsequent analyses.

### 2.4 LPS treatment

AC16 cardiomyocytes were starved for 12 h when it reached 80% confluence. Subsequently, the cells were stimulated with LPS (Beyotime, Shanghai, China) at a final concentration of 10 µg/mL for 12 h to induce an inflammatory response in cardiomyocytes.

### 2.5 Apoptosis assay

The level of cell apoptosis was measured using an Annexin V-PE/7-AAD Apoptosis Detection kit (Yeasen Biotechnology, Shanghai, China) according to the manufacturer’s instructions. AC16 cardiomyocytes were cultured in 6-well plates. When the cells reached 70% confluence, AC16 cardiomyocytes were transfected with IVT mRNA (0.1 μg/mL). Approximately 48 h after transfection, the cells were collected and washed twice with pre-cooled PBS at 4 °C. The supernatant was discarded, and the cells were resuspended in 1× binding buffer containing 50 μL/mL Annexin V-PE and 100 μL/mL 7-AAD. The cells were gently mixed and incubated at room temperature (25 °C) for 15 min in the dark. Subsequently, the level of cell apoptosis was detected using a BD FACSCalibur (BD Biosciences, Franklin Lakes, NJ, USA), and the data was analyzed with FlowJo 10 (VX.0.7) software (Tree Star, Ashland, OR, USA). The experiment was repeated in triplicates.

### 2.6 Reverse transcription–quantitative real-time polymerase chain reaction

Total RNA from AC16 cardiomyocytes was extracted using TRIzol™ Reagent (Invitrogen, Thermo Fisher Scientific, MA, USA) according to the manufacturer’s protocol. The concentration of the extracted RNA was measured using a NanoDrop™ 8000 spectrophotometer (Thermo Fisher Scientific, Waltham, MA, USA). cDNA was synthesized using a Reverse Transcription Kit (Takara, Osaka, Japan). RT-qPCR was performed using a SYBR Green PCR Master Mix on a QuantStudio™ 6 Flex System (Applied Biosystems, Foster City, MA, USA). Primer specificity was determined by melting curve analysis. Relative gene expression levels were calculated using the 2^-ΔΔCt^ method. Primer efficiencies were confirmed to be between 90% and 110% using standard curve analysis, validating the use of the ΔΔCt method for relative quantification (**Supplementary Figure S1**). β-actin was used as the internal control for mRNAs expression, and the expression levels of mature microRNAs (miRNAs) were normalized to U6. The quantification of miRNAs was based upon the stem-loop RT primer approach using a miRNA-specific primer to convert miRNA into cDNA, which was then amplified by a pair of qPCR primers. The detailed stem-loop primer sequences used for the reverse transcription of miRNAs are as follows: miR-149-5p: GTCGTATCCAGTGCAGGGTCCGAGGTATTCGCACTGGATACGACGGGAGT;

miR-98-5p: GTCGTATCCAGTGCAGGGTCCGAGGTATTCGCACTGGATACGACAACAAT; let-7a-5p: GTCGTATCCAGTGCAGGGTCCGAGGTATTCGCACTGGATACGACAACTAT;

let-7c-5p: GTCGTATCCAGTGCAGGGTCCGAGGTATTCGCACTGGATACGACAACCAT;

let-7f-5p: GTCGTATCCAGTGCAGGGTCCGAGGTATTCGCACTGGATACGACAACTAT.

The sequences of the primers used are listed in **Table 1**.

**Table 1:** Primers for RT-qPCR in this study.

Primers	Sequences (5’ to 3’)
β-actin	Forward: CACCATTGGCAATGAGCGGTTC
	Reverse: AGGTCTTTGCGGATGTCCACGT
IVT mRNA	Forward: TACGAGCAGTACATCAAGTG
	Reverse: TCATGTGTAGTGCAGTTTCA
U6	Forward: CTCGCTTCGGCAGCACAT
	Reverse: TTTGCGTGTCATCCTTGCG
miR-149-5p	Forward: AAGTCGCTCTGGCTCCGTGT
	Reverse: ATCCAGTGCAGGGTCCGAGG
miR-98-5p	Forward: CGCGCGTGAGGTAGTAAGTTGT
	Reverse: ATCCAGTGCAGGGTCCGAGG
let-7a-5p	Forward: CCACGGAGTGAGGTAGTAGGTTG
	Reverse: ATCCAGTGCAGGGTCCGAGG
let-7c-5p	Forward: CCACGGAGTGAGGTAGTAGGTTG
	Reverse: ATCCAGTGCAGGGTCCGAGG
let-7f-5p	Forward: CGCGCGTGAGGTAGTAGATTGT
	Reverse: ATCCAGTGCAGGGTCCGAGG
IL-6	Forward: ACTCACCTCTTCAGAACGAATTG
	Reverse: CCATCTTTGGAAGGTTCAGGTTG
U1	Forward: GGGAGATACCATGATCACGAAGGT
	Reverse: CCACAAATTATGCAGTCGAGTTTCCC
GAPDH	Forward: GTCTCCTCTGACTTCAACAGCG
	Reverse: ACCACCCTGTTGCTGTAGCCAA

### 2.7 Inflammatory cytokine assay

The Luminex liquid suspension chip was detected using the BioPlex protein array (Bio-Rad, Hercules, CA, USA) and the Luminex 200 detection system (Luminex Corporation, Austin, TX, USA) according to the manufacturers’ specifications. Upon the confluence of AC16 cardiomyocytes reached 70%, the cells were transfected with IVT mRNA (0.1 μg/mL). Approximately 48 h after transfection, the medium was then changed to FBS-free DMEM/F12 for an additional 24 h. The cell supernatant was subsequently collected for inflammatory cytokine analysis. The experiment was repeated in triplicates.

### 2.8 Enzyme-linked immunosorbent assay

The levels of Creatine Kinase-MB (CK-MB) and cardiac troponin I (cTN-I) in the supernatant of AC16 cardiomyocytes were measured using CK-MB (ml026270V; MLBio, Shanghai, China) and cTN-I ELISA kits (ml023547V; MLBio, Shanghai, China), respectively. Similarly, the interleukin-6 (IL-6) inflammatory cytokine content in the supernatant of AC16 cell cultures was determined using a human-specific IL-6 ELISA kit (Beijing 4A Biotech Co., Ltd., Beijing, China). When AC16 cardiomyocytes reached 70% confluence, the cells were transfected with IVT mRNA (0.1 μg/mL) for 48 h, followed by a medium change to FBS-free DMEM/F12 for 24 h. The cell supernatant was then collected for inflammatory cytokine analysis. The experiment was repeated in triplicates.

### 2.9 RNA immunoprecipitation

RIP assays were performed using the Magna RIP™ RNA-Binding Protein Immunoprecipitation Kit (Millipore, <ns/>17-700, USA) according to the manufacturer’s protocol. After the expansion of AC16 cardiomyocytes, the cells were collected and lysed in RIP lysis buffer containing RNase and protease inhibitors. The cell lysates were then incubated with rotation overnight at 4 °C with magnetic beads conjugated to 5 μg of rabbit anti-Ago2 (ab186733, Abcam, Cambridge, UK) and 5 μg of negative control rabbit IgG. On the following day, the beads were washed 10 times with wash buffer on the magnetic separator. The isolated RNA was then purified and precipitated, and the expression levels of target RNAs were subsequently determined by RT-qPCR. The experiment was repeated in triplicates.

### 2.10 Dual luciferase reporter assay

The GV272 (SV40-firefly_Luciferase-MCS) plasmid was used to construct the IVT mRNA-3’ UTR and IL-6 mRNA-3’ UTR luciferase reporter gene plasmid (GENECHEM Biotech, Shanghai, China). Wild−type (Wt) and mutant (Mt) 3’ UTRs of IVT mRNA and IL-6 mRNA were synthesized and cloned into the downstream sites of the GV272 vector (GENECHEM Biotech, Shanghai, China). AC16 cardiomyocytes were seeded into 12-well plates and co-transfected with Wt/Mt plasmids and miR-NC/miR-98-5p/let-7a-5p/let-7f-5p mimics (RiboBio, Guangzhou, China) using Lipo8000™ transfection reagent according to the manufacturer’s protocol. After the incubation for 48 h, the cells were harvested and lysed, and relative luciferase activity was measured using a Dual Luciferase Reporter Gene Assay Kit (Beyotime, Shanghai, China) according to the manufacturer’s protocol. The relative light units (RLU) of Firefly Luciferase and Renilla Luciferase of each sample were measured using a Centro LB 960 Microplate Luminometer (Berthold Technologies, Bad Wildbad, Germany). The RLU of Renilla Luciferase was used as an internal standard control. The relative luciferase activity of each sample was calculated as the RLU ratio of Firefly Luciferase/Renilla Luciferase. The experiment was repeated in triplicates.

### 2.11 Copy number analysis

1 × 10^6^ cells were utilized for copy number analysis. Absolute quantification was conducted using a series of 10-fold dilutions of the reference standard. The Ct values obtained from qPCR analysis were plotted against the dilution factors, and the data were fitted to a straight line. The copy numbers of IL-6 and let-7f-5p were determined based on the standard curve.

### 2.12 Statistical analysis

All experiments in this study were repeated in biological triplicates. Data are presented as the mean ± standard deviation (SD) and were analyzed using GraphPad Prism 9 software (GraphPad Software, San Diego, CA, USA). The Student’s t-test was used to determine statistical significance between two groups, while a one-way analysis of variance (ANOVA) was applied for comparisons among three or more groups. A *p*-value < 0.05 was considered statistically significant. Significance levels were indicated as follows: *p* < 0.05 (*), *p* < 0.01 (**), *p* < 0.001 (***), and *p* < 0.0001 (****). “ns” represents no significant difference.

## 3 Results

### 3.1 Primary structure of the Pfizer-BioNTech COVID-19 mRNA vaccine

**Figure 1:**
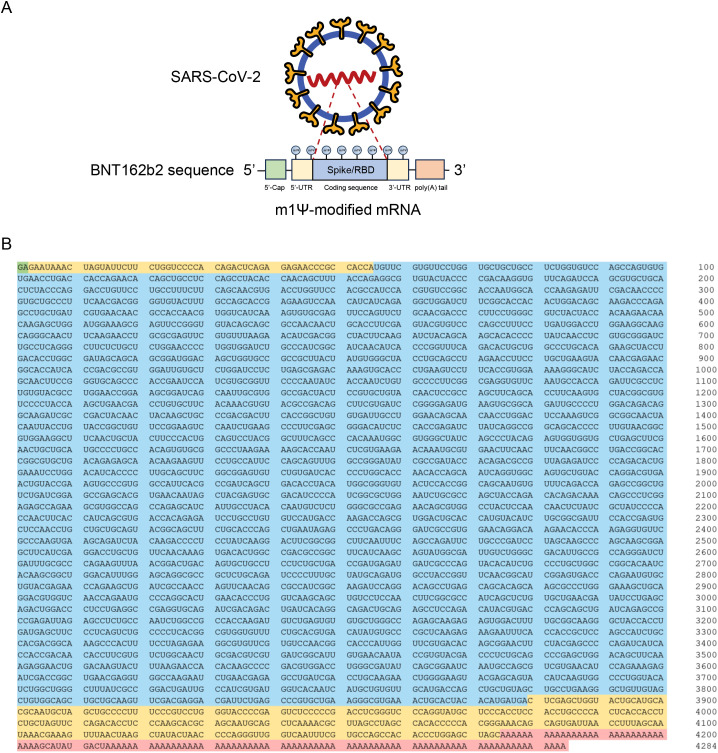
Design and encapsulation of synthetic COVID-19 mRNA encoding the SARS-CoV-2 RBD. **(A)** Design elements present in synthetic COVID-19 mRNA therapeutics. RBD: Receptor binding domain. **(B)** Sequence of the COVID-19 mRNA vaccine (BNT162b2) from Pfizer/BioNTech. Green: 5’-cap. Yellow: 5’- and 3’-UTR sequences. Blue: SARS-CoV-2 spike glycoprotein coding sequence. Red: Segmented poly(A) tail.

The sequence of the Pfizer-BioNTech COVID-19 mRNA vaccine (BNT162b2) has been previously disclosed (23). This 4284-nucleotide RNA construct comprises five key elements: 5’-cap, 5’ UTR, SARS-CoV-2 spike glycoprotein coding sequence, 3’ UTR, and poly(A) tail (**Figures 1A, B**).

### 3.2 IVT mRNA induces pro-inflammatory responses and cardiomyocyte apoptosis

**Figure 2:**
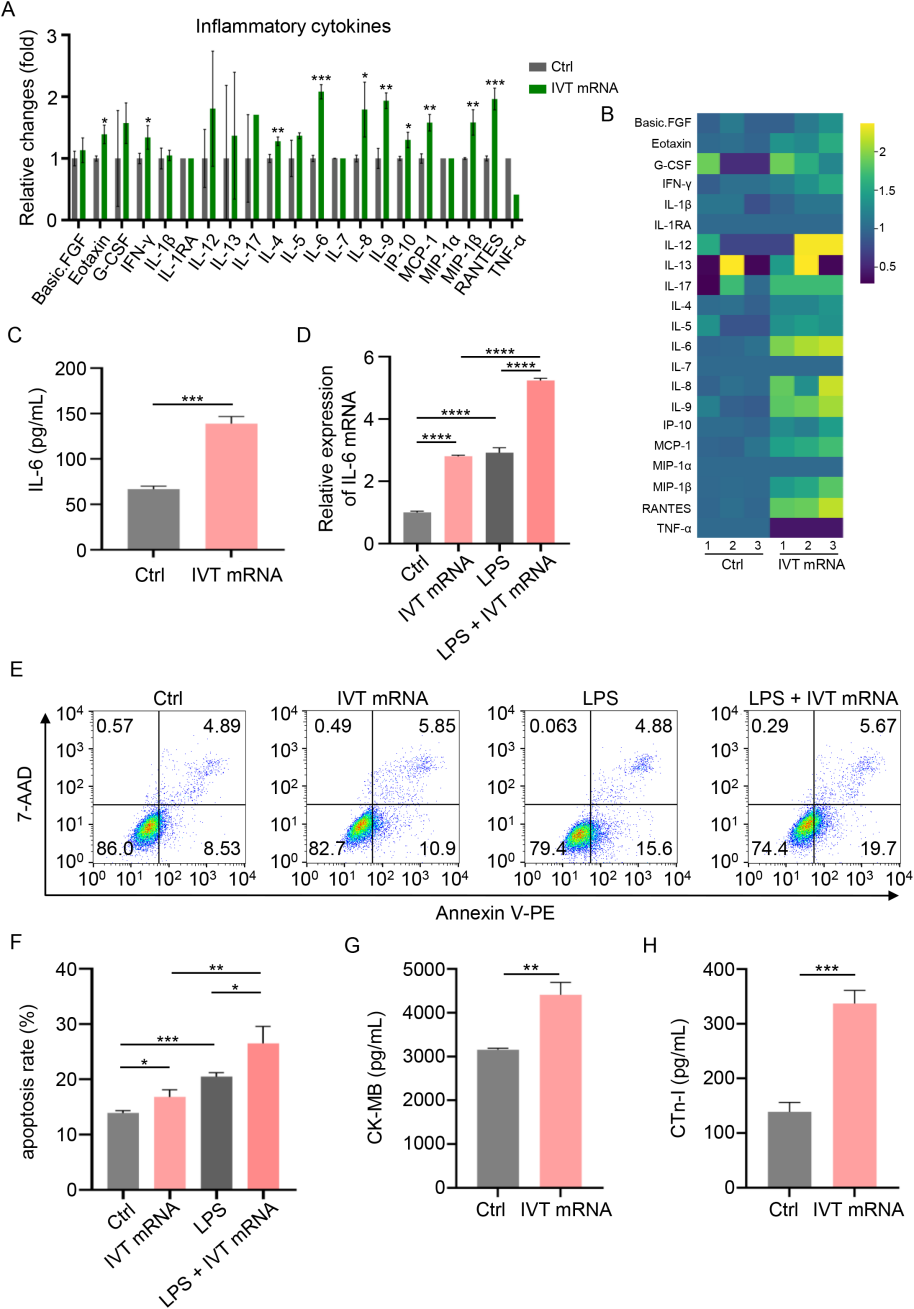
Inflammatory condition induced by IVT mRNA in AC16 cardiomyocytes. **(A)** Quantitative analysis of inflammatory cytokine production in the supernatant of cultured AC16 cardiomyocytes, assessed using a Luminex liquid suspension chip. **(B)** Heatmap of the production levels of the inflammatory cytokines in the cultured AC16 cardiomyocytes supernatant, assessed using a Luminex liquid suspension chip. **(C)** Quantification of IL-6 production levels in the same supernatant, determined by ELISA. **(D)** RT-qPCR analysis of IL-6 mRNA expression in AC16 cardiomyocytes stimulated with IVT mRNA, LPS, and LPS + IVT mRNA. **(E, F)** Quantitative analysis of the apoptosis rate in AC16 cardiomyocytes following treatment with IVT mRNA, LPS, and LPS + IVT mRNA. **(G, H)** Quantification of CK-MB, and cTn-I levels in the culture supernatant of AC16 cardiomyocytes, determined by ELISA. Statistical significance was determined using Student’s *t* test **(A, C, G, H)**, and One-way ANOVA **(D, F)**. *p < 0.05, **p < 0.01, ***p < 0.001, ****p < 0.0001 versus the control (Ctrl) group. The experiments were performed in triplicates, and data are presented as n = 3, mean ± SD.

To investigate the potential effects of IVT mRNA on inflammatory signaling in cardiomyocytes, we employed Luminex technology to quantify 21 inflammatory cytokines in AC16 cardiomyocyte supernatants. Cytokine levels were quantified (**Figure 2A**) and visualized as a heatmap (**Figure 2B**). Notably, IL-6 showed the most pronounced upregulation post-transfection. As a key mediator of the immune response, IL-6 is closely associated with various inflammatory diseases, including myocarditis (17, 18). ELISA results further validated the increase in IL-6 expression, consistent with Luminex chip results (**Figure 2C**). RT-qPCR experiments confirmed that IL-6 mRNA levels were significantly elevated in cardiomyocytes after IVT mRNA transfection (**Figure 2D**). Additionally, we induced an inflammatory response in the cells using LPS, and under inflammatory conditions, IVT mRNA further exacerbated IL-6 secretion (**Figure 2D**). To further explore the effects of IVT mRNA on AC16 cardiomyocyte function, we performed apoptosis assays. The results demonstrated that IVT mRNA transfection significantly promoted cardiomyocyte apoptosis. Furthermore, under LPS-induced inflammatory conditions, IVT mRNA exacerbated apoptosis in cardiomyocytes (**Figures 2E, F**). Following IVT mRNA transfection, there was a significant increase in the secretion of myocarditis markers CK-MB and cTn-I (**Figures 2G, H**). These findings indicated that IVT mRNA promoted the secretion of inflammatory cytokines and increased apoptosis in cardiomyocytes.

### 3.3 IVT mRNA serves as a sponge for let-7f-5p and IL-6 is a downstream target of let-7f-5p in AC16 cardiomyocytes

**Figure 3:**
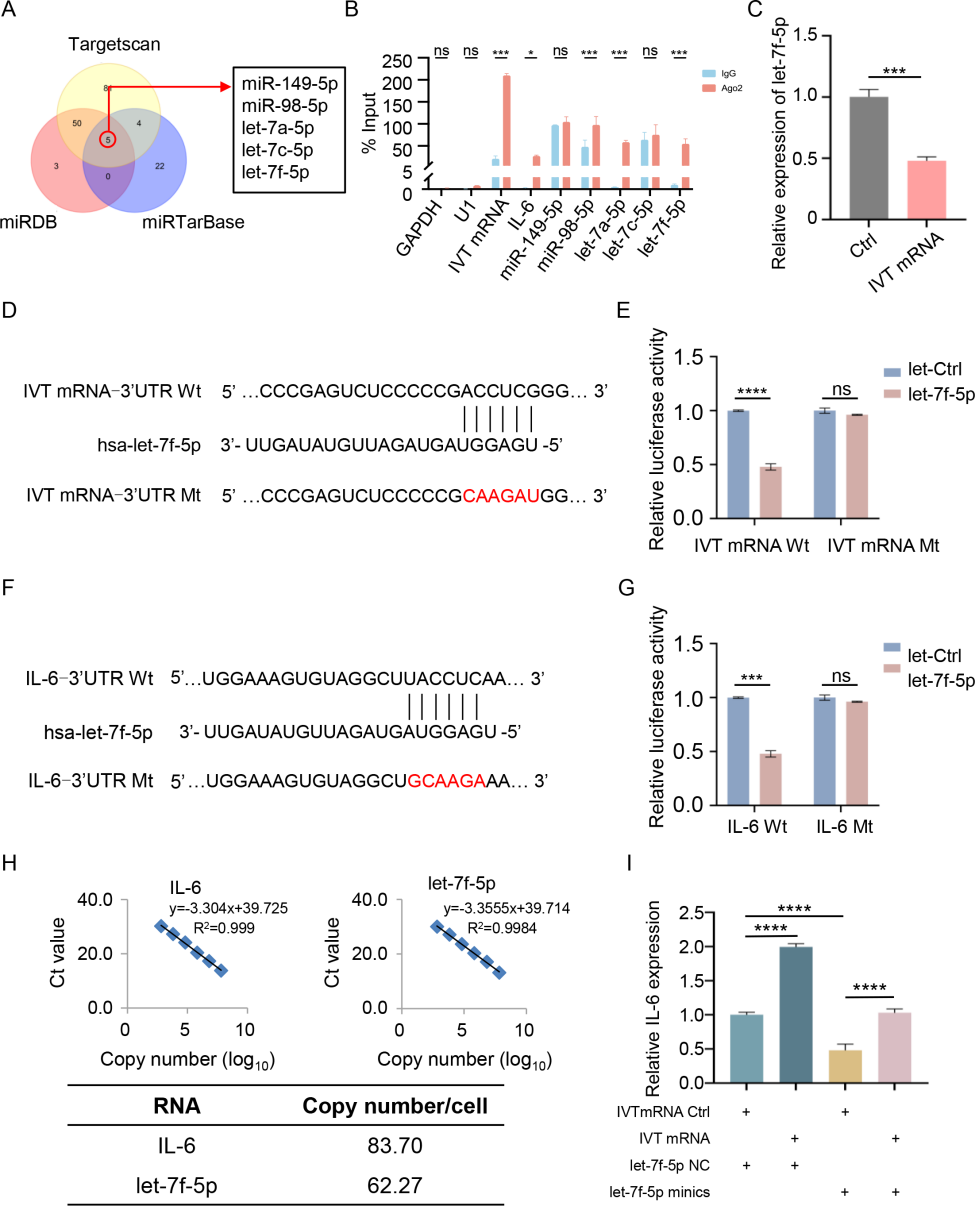
IVT mRNA functions as a miRNA sponge for let-7f-5p, regulating IL-6 expression. **(A)** Overlap of predicted IL-6 target miRNAs using TargetScan, miRDB, and miRTarBase. **(B)** RNA immunoprecipitation (RIP) analysis of IVT mRNA in AC16 cardiomyocytes using an anti-Ago2 antibody. **(C)** Relative expression levels of let-7f-5p in AC16 cardiomyocytes, measured by RT-qPCR. **(D)** Schematic illustration of the predicted let-7f-5p binding site on IVT mRNA. **(E)** Luciferase activity of wild-type and mutant IVT mRNAs in AC16 cardiomyocytes following co-transfection with let-7f-5p or a negative control miRNA. **(F)** Schematic illustration of the predicted let-7f-5p binding site on IL-6 mRNA. **(G)** Luciferase activity of wild-type and mutant IL-6 mRNAs in AC16 cardiomyocytes following co-transfection with let-7f-5p or a negative control miRNA. **(H)** Copy numbers of IL-6 and let-7f-5p in AC16 cells. Top left: linear correlation between log-transformed IL-6 copy number and Ct values as determined by RT-qPCR. Top right: linear correlation between log-transformed let-7f-5p copy number and Ct values. Bottom: gene copy number analysis in AC16 cells. **(I)** Relative mRNA levels of IL-6 in AC16 cardiomyocytes transfected with IVT mRNA Ctrl, IVT mRNA, let-7f-5p NC, or let-7f-5p mimics, as measured by RT-qPCR. Statistical significance was determined using Student’s *t* test (B, C, E, and G), and One-way ANOVA **(I)**. *p < 0.05, ***p < 0.001, ****p < 0.0001, “ns” indicates no significant difference. The experiments were performed in triplicates, and data are presented as n = 3, mean ± SD.

**Figure 4:**
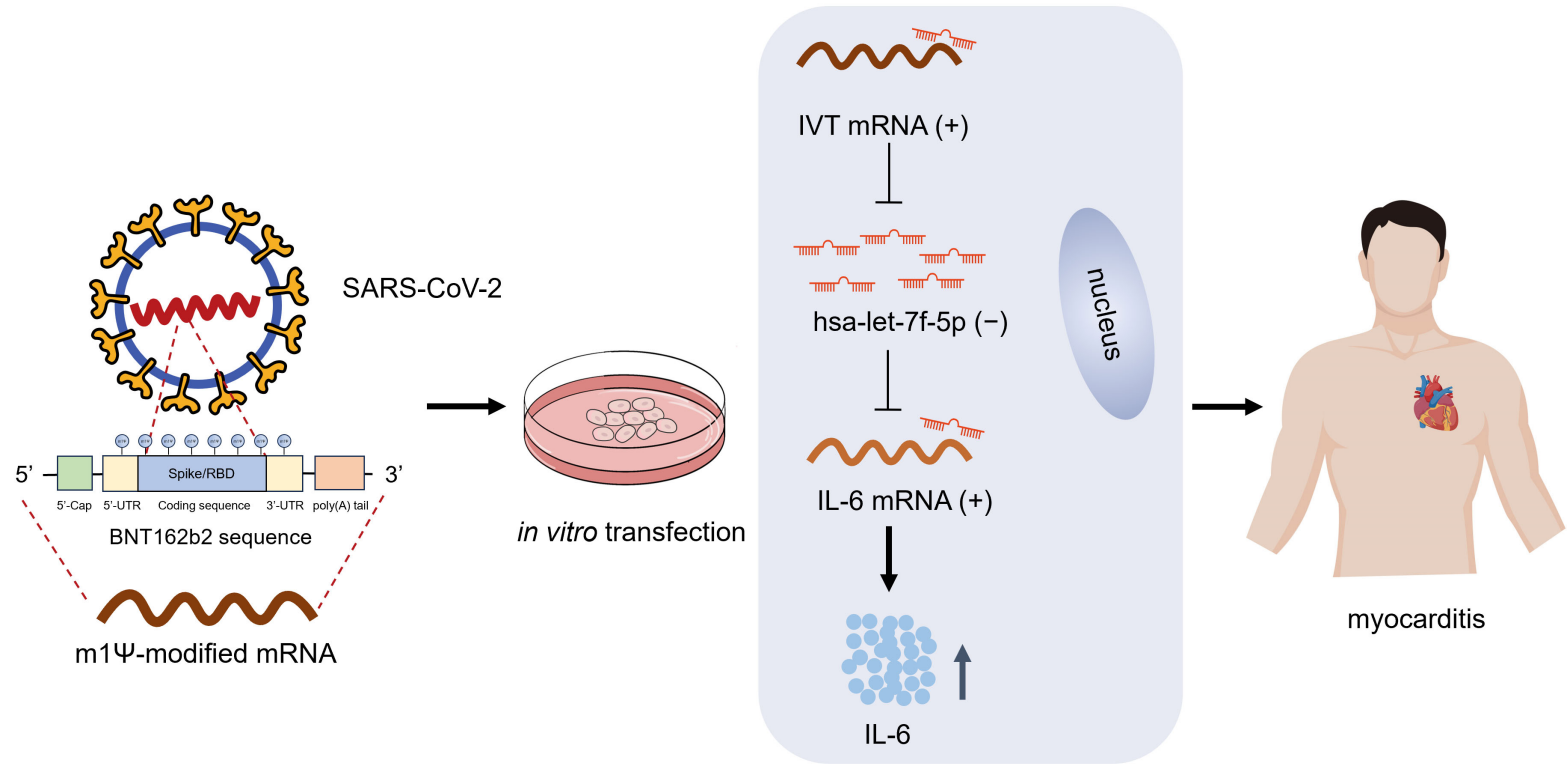
Working model of IVT mRNA function in myocarditis. Human AC16 cardiomyocytes were transfected with *in vitro* transcribed (IVT) COVID-19 mRNA incorporating N1-methyl-pseudouridine (m1Ψ) modifications. Within cardiomyocytes, the IVT mRNA functions as a ceRNA, sequestering let-7f-5p through a sponge effect. This interaction relieves the post-transcriptional repression of IL6, leading to increased IL6 expression and subsequent induction of myocardial inflammation.

Given the enrichment of IVT mRNA in the cytoplasm, we hypothesized that it might function as a ceRNA to regulate IL-6 translation by sponging specific miRNAs implicated in the pathogenic process of myocarditis. To test this hypothesis, we employed three publicly available prediction tools—TargetScan, miRDB, and miRTarBase—to identify potential miRNAs that could bind to the IL-6 mRNA-3’ UTR. These analyses predicted five candidate miRNAs (miR-149-5p, miR-98-5p, let-7a-5p, let-7c-5p, and let-7f-5p) with binding potential to the IL-6 mRNA-3’ UTR (**Figure 3A**). Using the RNAhybrid database, we further confirmed that these miRNAs could also bind to the IVT mRNA-3’ UTR. To experimentally validate these interactions, RNA immunoprecipitation (RIP) assays were performed in AC16 cardiomyocytes. The results demonstrated that miR-98-5p, let-7a-5p, let-7f-5p, IVT mRNA, and IL-6 mRNA were co-immunoprecipitated with an anti-Ago2 antibody (**Figure 3B**), suggesting functional interactions between IVT mRNA, IL-6 mRNA, and these miRNAs. Previous studies have demonstrated that hsa-let-7f-5p directly regulates IL-6 expression in radiation enteritis (24). RT-qPCR showed that let-7f-5p expression decreased after transfection with IVT mRNA (**Figure 3C**). To confirm this regulatory role, we constructed luciferase reporter vectors containing wild-type (Wt) or mutant (Mt) binding sites for let-7f-5p in the 3’ UTRs of IVT mRNA and IL-6 mRNA (**Figures 3D, F**). Transfection of let-7f-5p mimics significantly reduced the luciferase activity of the wild-type IVT mRNA-3’ UTR reporter in AC16 cardiomyocytes (**Figure 3E**). A similar reduction in luciferase activity was observed for the wild-type IL-6 mRNA-3’ UTR reporter under the same conditions (**Figure 3G**). However, no significant changes in luciferase activity were detected when mutant IVT mRNA or IL-6 mRNA-3’ UTR reporters were co-transfected with let-7f-5p mimic or a scrambled control miRNA. A fundamental requirement for investigating ceRNA mechanisms is that the copy numbers of mRNA and microRNA are comparable. To support the ceRNA hypothesis, we measured the copy numbers of IL-6 and let-7f-5p in AC16 cells using absolute quantification. The results revealed comparable copy numbers, with each AC16 cell containing approximately 83.70 copies of IL-6 and 62.27 copies of let-7f-5p (**Figure 3H**). Furthermore, RT-qPCR analysis demonstrated that IVT mRNA transfection significantly increased IL-6 expression, while let-7f-5p mimics significantly reduced it (**Figure 3I**). These findings indicate that IL-6 expression is regulated by let-7f-5p and IVT mRNA through a ceRNA mechanism, providing insight into the molecular interactions underlying myocarditis associated with COVID-19 mRNA vaccines (**Figure 4**).

## 4 Discussion

As one of the leading technologies in the development of COVID-19 vaccines, mRNA vaccines have several advantages over traditional vaccines. These advantages include faster production, higher specificity in targeting viral antigens, and the absence of live pathogens (25). Moreover, their flexible design allows for rapid adaptation to new viral variants. However, in addition to potentially triggering an immune response as an exogenous antigen like traditional vaccines, mRNA vaccines may activate immune reactions through other mechanisms. Our findings revealed that IVT mRNA with sequences identical to those used in COVID-19 vaccines triggered robust inflammation in AC16 cardiomyocytes, as evidenced by the significant upregulation of IL-6 and other inflammatory cytokines. Disease Ontology enrichment analysis of AC16 cardiomyocytes further showed that the differentially expressed genes were associated with cardiovascular diseases. Additionally, our study suggested that IVT mRNA acted as a ceRNA, specifically sponging let-7f-5p to regulate IL-6 expression. This ceRNA activity was validated through bioinformatics predictions, RNA immunoprecipitation (RIP) assays, and luciferase reporter assays, highlighting the interplay between IVT mRNA, let-7f-5p, and IL-6 in cardiomyocytes. These findings provide valuable insights into the molecular mechanisms underlying IVT mRNA-induced inflammation and its potential implications for cardiovascular health.

Our study aligns with and extends previous research on the inflammatory effects of mRNA-based therapeutics and vaccines. Prior studies have documented the pro-inflammatory properties of mRNA vaccines, particularly in the context of COVID-19, where mRNA vaccination has been associated with myocarditis (11, 16). Moreover, myocarditis has been reported more frequently in males and individuals under the age of 30 following the second dose of the vaccine (26, 27). These studies suggested that vaccines may induce or exacerbate inflammatory responses in some populations, including those with pre-existing inflammatory conditions, which could heighten the risk of adverse reactions (28). In addition, recent reports have described increased IL-6 production in individuals who received COVID-19 mRNA vaccines. Langgartner et al. reported that PBMCs from vaccinated donors released significantly higher levels of IL-6 *ex vivo* compared with baseline (29). Similarly, Alghamdi et al. observed altered circulating cytokine profiles, including elevated IL-6, in healthy individuals following mRNA vaccination (30). Furthermore, Miyashita et al. demonstrated that serum IL-6 levels were elevated after COVID-19 mRNA vaccination, particularly following the second dose. These clinical observations are consistent with our findings and highlight the potential contribution of IL-6–mediated inflammation to COVID-19 mRNA vaccine-associated myocarditis (31). Therefore, there is growing concern about the safety of mRNA vaccines in individuals with underlying inflammatory conditions. We speculate that mRNA vaccines could theoretically exacerbate the inflammatory response in individuals who already have myocardial damage, as their heart tissue may be in a vulnerable or compromised state. Because mRNA vaccines may trigger a local or systemic inflammatory response, there may be a risk of further aggravating inflammation or myocarditis in these individuals.

N1-methylpseudouridine (m1Ψ) is a critical chemical modification in COVID-19 mRNA vaccines, significantly enhancing their safety and efficacy. Early studies demonstrated that m1Ψ, like unmodified uridine, does not markedly affect codon decoding accuracy during protein synthesis. Incorporation of m1Ψ into mRNA did not increase miscoded peptides in cell culture, nor did it stabilize mismatched RNA duplexes, thereby preserving translation fidelity (32). However, a recent *Nature* study revealed that m1Ψ induces +1 ribosomal frameshifting during translation due to ribosome stalling at specific “slippery sequences,” generating unintended protein products capable of triggering off-target immune responses (33). The primary advantage of m1Ψ lies in its reduced immunogenicity: unmodified mRNA activates innate immune sensors (e.g., TLRs), leading to mRNA degradation and reduced efficacy, whereas m1Ψ-modified mRNA evades immune recognition, enhancing stability and protein expression (34, 35). Furthermore, m1Ψ increases mRNA resistance to ribonuclease degradation, prolonging intracellular persistence and enabling sustained protein production (36). Notably, while frameshifted products are minor components (~10% of total translation) and no adverse effects have been clinically reported, this discovery highlights an underappreciated risk in mRNA design—distinct from but potentially additive to ceRNA-mediated immunogenicity.

The ceRNA hypothesis proposed in this study extends the paradigm of RNA crosstalk, demonstrating that exogenous IVT mRNA—like endogenous mRNAs, lncRNAs, and circRNAs (37)—can competitively sequester miRNAs (e.g., let-7f-5p) to regulate disease-associated genes (e.g., IL-6). While canonical miRNA-mRNA studies focus on 3’ UTR interactions, our findings highlight a broader implication: therapeutic mRNA sequences, even when optimized for translation efficiency and immunogenicity, may inadvertently harbor miRNA response elements (MREs) that trigger ceRNA-mediated off-target effects. Although the impact of chemical modifications (e.g., m1Ψ) on miRNA binding remains uncharacterized, recent advances in nucleotide-modified mRNA therapeutics, such as those reported in *Nature (* 33), underscore the need to evaluate how RNA modifications alter not only translation but also RNA interactome dynamics—a frontier our study directly addresses. To mitigate risks, we propose integrating MRE avoidance algorithms into current mRNA design pipelines, particularly for vaccines targeting populations with pre-existing inflammatory conditions. Future work should establish *in vivo* ceRNA validation platforms and explore chemical modifications that selectively disrupt unintended miRNA interactions without compromising translational efficacy.

Several limitations of this study should be acknowledged, which also point to avenues for future research. First, all experiments were conducted in the human AC16 cardiomyocyte cell line, which may not fully capture the complexity of the *in vivo* cardiac microenvironment, including interactions with immune cells and systemic factors. Second, the ceRNA mechanism between IVT mRNA and hsa-let-7f-5p was demonstrated *in vitro*, and its relevance to *in vivo* myocarditis or vaccine-associated adverse events remains to be validated in animal models and clinical samples. Although direct measurements of let-7a-5p and let-7f-5p expression in cardiac tissue from COVID-19 mRNA–vaccinated mice have not yet been reported, previous studies support the potential relevance of these miRNAs in cardiac inflammatory responses. For instance, Atherton et al. observed dynamic regulation of let-7f-5p in murine immune response models, suggesting its role in modulating inflammation (38). Additionally, Bernstein et al. reviewed the involvement of let-7 family microRNAs in cardiovascular diseases and inflammation, highlighting their potential functional roles in the heart (39). These findings provide indirect evidence supporting the biological plausibility of let-7a-5p/let-7f-5p participation in mRNA vaccine–induced myocardial inflammation and justify further *in vivo* investigation. Together, these observations suggest that our *in vitro* results may reflect potential *in vivo* effects, but direct validation in animal models or patient-derived cardiac tissue will be critical to confirm the physiological relevance of the ceRNA-mediated IL-6 regulation. Finally, our analyses were limited to short-term cellular responses following transfection, and the potential long-term or cumulative effects of mRNA exposure on cardiomyocytes were not assessed. Future studies employing *in vivo* models, patient-derived samples, and systems-level approaches will be essential to confirm and extend these findings.

## 5 Conclusion

Our study reveals that exogenous mRNA vaccines can inadvertently dysregulate endogenous gene networks via ceRNA competition, exemplified by let-7f-5p-mediated IL-6 overexpression. This discovery challenges the conventional view of therapeutic mRNA as a passive antigen template, revealing its dual role as both an antigen blueprint and a post-transcriptional regulator. To ensure translational fidelity and biological safety, future mRNA vaccines must adopt sequence designs that minimize miRNA sponge activity, thereby advancing precision RNA therapeutics.

## Data Availability

The original contributions presented in the study are included in the article/**Supplementary Material**. Further inquiries can be directed to the corresponding author.
