# Supplementary material for: Exploring ceRNA mechanisms in COVID-19 mRNA vaccine-induced myocarditis: implications for future vaccine design
Source: Front Immunol. 2025 Oct 22;16:1674049. doi: 10.3389/fimmu.2025.1674049 (PMC12586086; doi:10.3389/fimmu.2025.1674049)
Supplement: Supplementary file 1 [file DataSheet1.pdf]

Exploring ceRNA Mechanisms in COVID-19 mRNA Vaccine-Induced Myocarditis: Implications for Future Vaccine Design

Jing Wang[1, 2, 3, 4, #], Xin-Yi Sun[4, #], Qian Gao[4], Min Fu[4], Mian Xiao[4], Ning Du[1], Xi-Yuan Ge[1, 2, 3, 4, *]

[1] Central Laboratory, Peking University School and Hospital of Stomatology, Beijing, 100081, PR China.

[2] National Center for Stomatology & National Clinical Research Center for Oral Diseases & National Engineering Research Center of Oral Biomaterials and Digital Medical Devices, Beijing, 100081, PR China

[3] Beijing Key Laboratory of Digital Stomatology, Beijing, 100081, PR China

[4] Department of Oral and Maxillofacial Surgery, Peking University School and Hospital of Stomatology, Beijing 100081, PR China.

[#] These authors contributed equally to this work.

* Corresponding Authors:

Xi-Yuan Ge, Peking University School and Hospital of Stomatology, No.22, Zhongguancun South Avenue, Haidian District, Beijing, 100081, PR China.

Tel:86-10-8219-5132; Fax:86-10-6217-9977; E-mail: gexiyuan@bjmu.edu.cn

**U1**	
Log (Concentration)	Ct
0	12.805
-1	15.896
-2	18.565
-3	21.574
-4	25.946

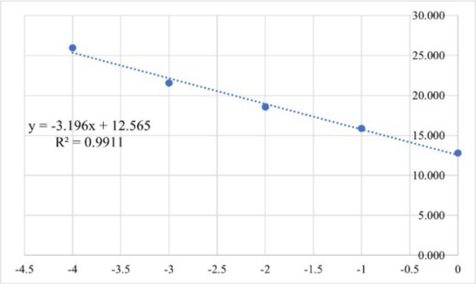

**U6**	
Log (Concentration)	Ct
0	16.100
-1	19.065
-2	21.569
-3	24.487
-4	28.994

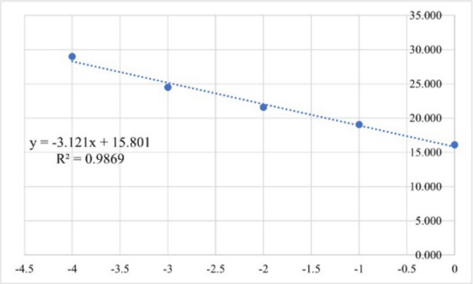

**IVT mRNA**	
Log (Concentration)	Ct
0	12.841
-1	15.630
-2	18.153
-3	20.982
-4	25.694

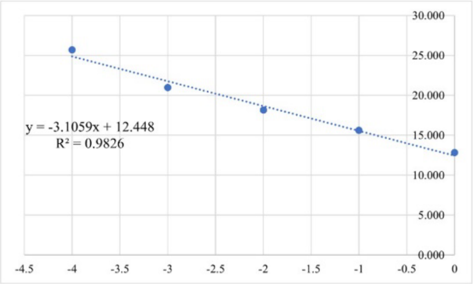

**IL-6**	
Log (Concentration)	Ct
0	23.059
-1	26.599
-2	29.144
-3	32.811
-4	35.493

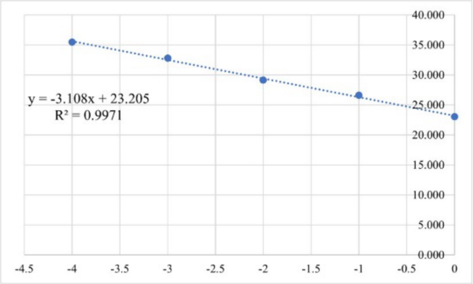

**GAPDH**	
Log (Concentration)	Ct
0	12.649
-1	15.829
-2	18.409
-3	21.286
-4	26.127

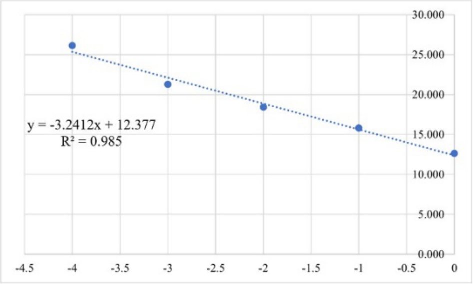

**β-actin**	
Log (Concentration)	Ct
0	11.720
-1	14.965
-2	17.498
-3	20.528
-4	25.183

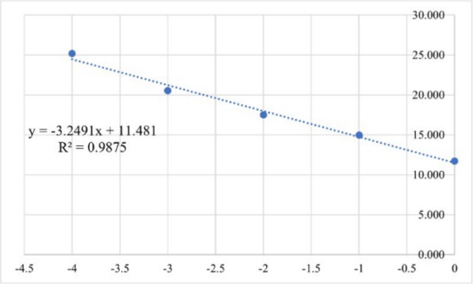

**miR-149-5p**	
Log (Concentration)	Ct
0	21.542
-1	24.898
-2	29.510
-3	31.432
-4	34.125

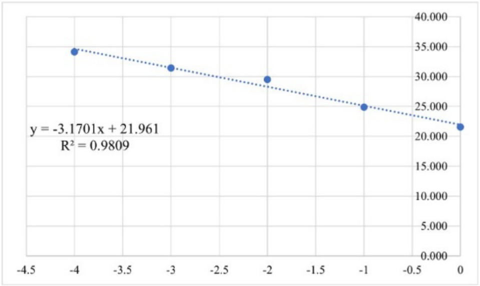

**miR-98-5p**	
Log (Concentration)	Ct
0	25.459
-1	29.427
-2	32.489
-3	34.974
-4	38.894

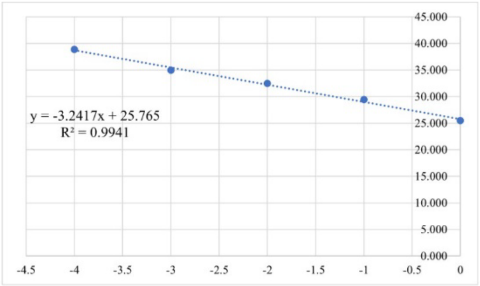

**let-7a-5p**	
Log (Concentration)	Ct
0	18.054
-1	22.291
-2	24.560
-3	27.376
-4	31.033

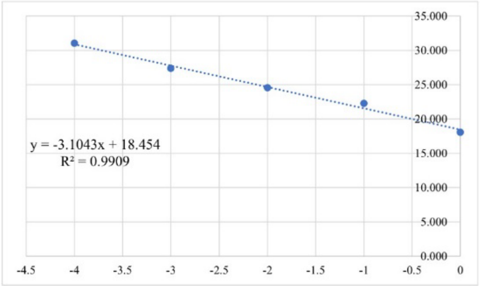

**let-7c-5p**	
Log (Concentration)	Ct
0	25.609
-1	29.764
-2	32.886
-3	35.374
-4	38.522

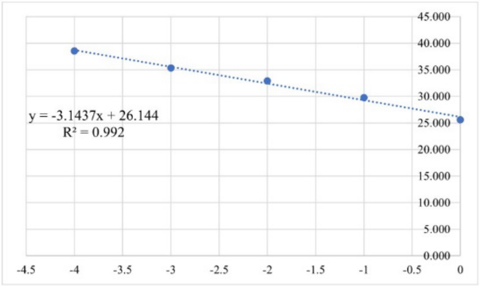

**let-7f-5p**	
Log (Concentration)	Ct
0	23.251
-1	27.247
-2	30.402
-3	33.623
-4	36.499

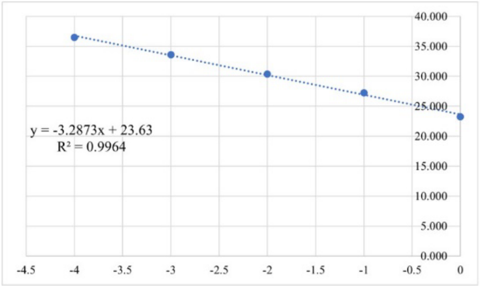

Figure S1. Standard curves for qPCR primer efficiency validation. Standard curves were generated for each primer set (U1, U6, IVT mRNA, IL-6, GAPDH, β-actin, miR-149-5p, miR-98-5p, let-7a-5p, let-7c-5p, and let-7f-5p) by plotting Ct values against the log of input cDNA concentration. Linear regression equations and R^2 values are shown in each panel.
